# Unveiling the relationship between WWOX and BRCA1 in mammary tumorigenicity and in DNA repair pathway selection

**DOI:** 10.1038/s41420-024-01878-8

**Published:** 2024-03-18

**Authors:** Tirza Bidany-Mizrahi, Aya Shweiki, Kian Maroun, Lina Abu-Tair, Bella Mali, Rami I. Aqeilan

**Affiliations:** 1 The Concern Foundation Laboratories, The Lautenberg Center for Immunology and Cancer Research, Department of Immunology and Cancer Research-IMRIC, Faculty of Medicine, The Hebrew University of Jerusalem, Jerusalem, Israel; 2 Department of Pathology, Hadassah University Hospital, Jerusalem, Israel; 3 Cyprus Cancer Research Institute (CCRI), Nicosia, Cyprus

**Keywords:** Breast cancer, Breast cancer

## Abstract

Breast cancer is the leading cause of cancer-related deaths in women worldwide, with the basal-like or triple-negative breast cancer (TNBC) subtype being particularly aggressive and challenging to treat. Understanding the molecular mechanisms driving the development and progression of TNBC is essential. We previously showed that WW domain-containing oxidoreductase (WWOX) is commonly inactivated in TNBC and is implicated in the DNA damage response (DDR) through ATM and ATR activation. In this study, we investigated the interplay between WWOX and BRCA1, both frequently inactivated in TNBC, on mammary tumor development and on DNA double-strand break (DSB) repair choice. We generated and characterized a transgenic mouse model (*K14-Cre;Brca1*^*fl/fl*^*;Wwox*^*fl/fl*^) and observed that mice lacking both WWOX and BRCA1 developed basal-like mammary tumors and exhibited a decrease in 53BP1 foci and an increase in RAD51 foci, suggesting impaired DSB repair. We examined human TNBC cell lines harboring wild-type and mutant BRCA1 and found that WWOX expression promoted NHEJ repair in cells with wild-type BRCA1. Our findings suggest that WWOX and BRCA1 play an important role in DSB repair pathway choice in mammary epithelial cells, underscoring their functional interaction and significance in breast carcinogenesis.

## Introduction

Breast cancer is the most common malignancy in women and the second leading cause of death [1, 2]. Triple-negative breast cancer subtype (TNBC) is a heterogeneous disease characterized by the absence of estrogen receptor (ER), progesterone receptor (PR), and wild-type human epidermal growth factor receptor 2 (HER2) with no amplification or overexpression. It is the most aggressive breast cancer subtype and known to exhibit poor prognosis due to its lack of targetable receptors. Basal-like breast cancers (BLBCs) represent a subset of about 70% of TNBCs [3]. TNBC is typically observed (75%) in women who carry a mutation in the Breast cancer type 1 susceptibility protein, *BRCA1*, gene [4]. Hereditary and sporadic BRCA1-associated breast cancers are often triple-negative and express basal markers [5], such as keratin 14 (K14) and K5.

BRCA1 is involved in the maintenance of genomic integrity and mainly relies on its central role in protein complexes that are required for the repair of double-strand breaks (DSB) and stalled replication forks [6]. *BRCA1* mutation carriers are at a higher risk of developing breast, ovarian, prostate, and other types of cancer. Furthermore, breast cancers with *BRCA1* mutations have earlier onset, more aggressive behavior, and a higher risk of recurrence [7]. Several functional partners of BRCA1 have been described, contributing to better characterization of BRCA1’s role in DSBs’ repair and its significance in breast carcinogenesis [1, 8–10].

DSBs are the most deleterious breaks in the genome [11, 12]. There are two main pathways for DNA DSB repair, homology-directed repair (HDR) and non-homologous end-joining (NHEJ). HDR is an error-free approach, as the break is fixed by homology with the adjacent sister chromatid. However, this pathway is limited to the S/G2 phase of the cell cycle, where chromosomes are duplicated into two sister chromatids. On the other hand, the NHEJ repair joins the two ends of a DSB directly, making it more error-prone, yet available for use throughout the entire cell cycle [13]. These pathways largely operate in complementary ways when repairing DSBs [14].

DSB repair pathway choice is tightly regulated and has been linked to cancer progression [11, 15, 16]. It has been proposed that during the S phase, when BRCA1 becomes more abundant, it forms a complex with CtIP, displacing RIF1/53BP1. Under these circumstances, BRCA1 binds the MRN complex, which triggers end resection. It is only then that RAD51 filaments on 3’ single strands are formed by BRCA2 and followed by strand invasion into homologous DNA [7]. In the absence of functional BRCA1, cells experience defects in HDR and are forced to rely on alternative DNA repair mechanisms. Such alteration can potentially lead to genomic instability, further increasing the risk of developing breast cancer [10, 17–21]. It should be noted that many critical factors other than BRCA1 including helicases (BLM) and nucleases (MRE11, EXO1, and DNA2) have been implicated in end resection however, others such as CtIP, for example, has been argued to have indispensable roles in promoting resection [22] hence highlighting the complexity of HDR regulation and the importance of in-depth understanding of its molecular basis.

WW domain-containing oxidoreductase (*WWOX*) encodes a 46-kDa tumor suppressor that is commonly inactivated in TNBC [23]. WWOX, as an adapter protein, has two WW domains, which mediate its interaction with proline-rich motifs-containing proteins [24]. Through physical interaction, WWOX has been shown to modulate and regulate function of a number of key breast cancer relevant proteins including ErbB4, AP2, p53, c-JUN, JNK1, DVL and MERIT40 [18, 24–33]. Anti-tumor activity of WWOX was also reported using mouse models as conditional deletion of *Wwox* in *C3H:MMTV-Cre* mice leads to spontaneous basal-like mammary tumor development exhibiting p53 inactivation [27, 28]. Whether WWOX cooperates with BRCA1 to suppress BLBCs and TNBCs is unknown.

An emerging and central function of WWOX that has been recently reported by several research groups is its direct role in the DNA damage response (DDR) and its involvement in DNA repair [18, 34]. We previously reported that WWOX enhances efficient repair of DSBs and DDR via interaction and regulation of ATM, a major DNA damage checkpoint protein [35, 36] as well as repair of single strand breaks (SSB) via activation of ATR [37]. More recently, WWOX binding with BRCA1, through the WW1 domain, was revealed. This interaction disrupts BRCA1 binding to the MRN complex, causing less DNA end resection, hence redirecting the cell to repair DSBs via NHEJ pathway rather than HDR [17, 38, 39]. In other words, WWOX expression shifts DSB repair from HDR to NHEJ. However, the consequence of WWOX-BRCA1 association and its loss have not been demonstrated in vivo using mouse models.

Since both BRCA1 and WWOX are commonly inactivated in TNBC and are involved in DNA repair, we set to test whether inactivation of both proteins would affect mammary tumorigenesis in vivo. Furthermore, we tested the effect of WWOX expression on DNA DSB repair pathway choice when *BRCA1* is either wild-type or mutant in human TNBC cell lines. Our findings demonstrate a functional interaction between WWOX and BRCA1, affecting DSB repair pathway choice and regulating mammary tumorigenesis.

## Results

### Combined targeted loss of *Wwox* and *Brca1* in K14+ cells result in basal-like mammary tumors in vivo

**Fig. 1:**
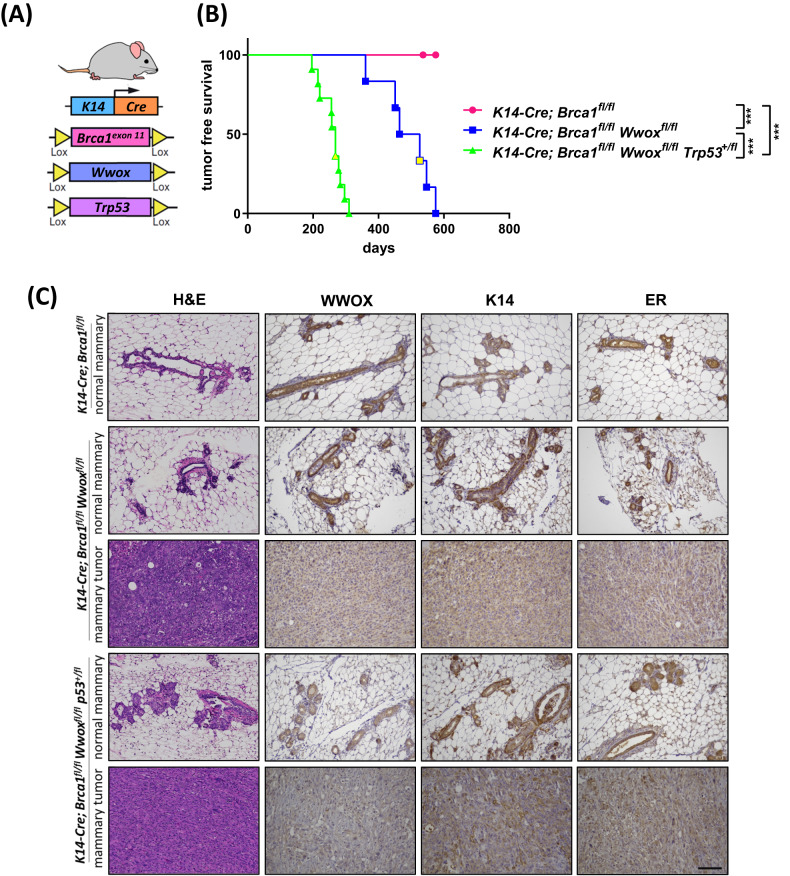
Combined loss of *Wwox* and *Brca1* results in basal- like mammary tumors in vivo. **A** The *K14-Cre;Brca1*^*fl/fl*^*Wwox*^*fl/fl*^*Trp53*^*+/fl*^ mice transgenic mouse model. *K14-Cre* conditionally expressed in the basal mammary epithelia, and basal epidermis. **B** Tumor frees survival curve of *K14-Cre;Brca1*^*fl/fl*^ (*n* = 6), *K14-Cre;Brca1*^*fl/fl*^*Wwox*^*fl/fl*^ (*n* = 6) and *K14-Cre;Brca1*^*fl/fl*^*Wwox*^*fl/fl*^*Trp53*^*+/fl*^ (*n* = 10) mice. Solid color- basal-like mammary tumor, yellow- cutaneous squamous cell carcinoma. Statistical significance calculated by Log-rank (Mantel-Cox) test. **C** H&E stain and IHC stain (WWOX, K14, ER) of normal mammary and mammary tumors from *K14-Cre;Brca1*^*fl/fl*^, *K14-Cre;Brca1*^*fl/fl*^*Wwox*^*fl/fl*^ and *K14-Cre;Brca1*^*fl/fl*^*Wwox*^*fl/fl*^ mice. 40×. Scale- 200 µm.

In a previous work, we observed that conditional deletion of *Wwox* in *C3H:MMTV-Cre* mice resulted in the development of basal-like mammary tumors [27, 28]. The majority of those mice developed tumors and display inactivation or downregulation of p53 [27, 28]. Given that WWOX and BRCA1 have been implicated in BLBC/TNBC and previously reported as interacting partners [17, 38, 39], we tested whether their targeted loss synergizes to affect mammary tumorigenesis in vivo. For this reason, we crossed the *K14-Cre* transgenic line with *Brca1* exon 11 flanked flox mice (referred to as *Brca1*^*fl/fl*^), to generate a conditional mutated mouse for *Brca1* in mammary basal cells (Fig. 1A); validation of transgene manipulation is shown in Supplementary Fig. 1. For the sake of simplicity in terminology, we referred to progeny of *K14-Cre*;*Brca1*^*fl/fl*^ mice as Brca1 KO. In this mouse model, mammary tumors only arise at low frequency and with very long latency, unless additional mutations in genes such as *Trp53* are present [8]. Indeed, consistent with published data, *K14-Cre*;*Brca1*^*fl/f*^ female mice did not give rise to mammary tumors until 800 days of age (Fig. 1B). On the other hand, *Wwox* ablation in mixed B6-129 genetic background also didn’t result in mammary tumor formation, consistent with previously published data [40, 41]. We next explored whether ablation of tumor suppressor *Wwox* can enhance mammary tumor formation in *K14-Cre;Brca1*^*fl/fl*^ mice. As expected, all *K14-Cre*;*Brca1*^*fl/fl*^*;Wwox*^*fl/fl*^ female mice developed basal-like mammary tumors with a median of 495 days (Fig. 1B). Additional depletion of one *Trp53* allele (*Trp53*^*+/fl*^) in the *K14-Cre*;*Brca1*^*fl/fl*^*;Wwox*^*fl/fl*^ mice reduced the median of tumor development to 268 days (Fig. 1B), without affecting tumor morphology (Fig. 1C). Both *K14-Cre*;*Brca1*^*fl/fl*^*;Wwox*^*fl/fl*^ and *K14-Cre*;*Brca1*^*fl/fl*^*;Wwox*^*fl/fl*^;*Trp53*^*+/fl*^ mammary tumors exhibited a basal-like morphology with occasional expression of K14 [42] and lack of ER expression (Fig. 1C), suggesting a worse prognosis recapitulating human BLBC/TNBC. Although normal mammary epithelium of these two mouse models expressed WWOX in most cells, conditional ablation of the indicated genes was enough for tumor development (Fig. 1C, Supplementary Fig 1). This is likely due to the mosaic *Cre* expression in this model where only 5–30% of mammary-gland epithelial cells express Cre [9] (Fig. 1C). The mammary tumors developed in these mice were indeed negative for WWOX expression (Fig. 1C). These results imply that WWOX has an important role in protecting against development of mammary tumors in which *Brca1* deficiency is involved.

### *K14-Cre;Brca1*^*fl/fl*^*;Wwox*^*fl/fl*^ mice mammary epithelia express less NHEJ and more HDR markers as compared to *K14-Cre;Brca1*^*fl/fl*^

**Fig. 2:**
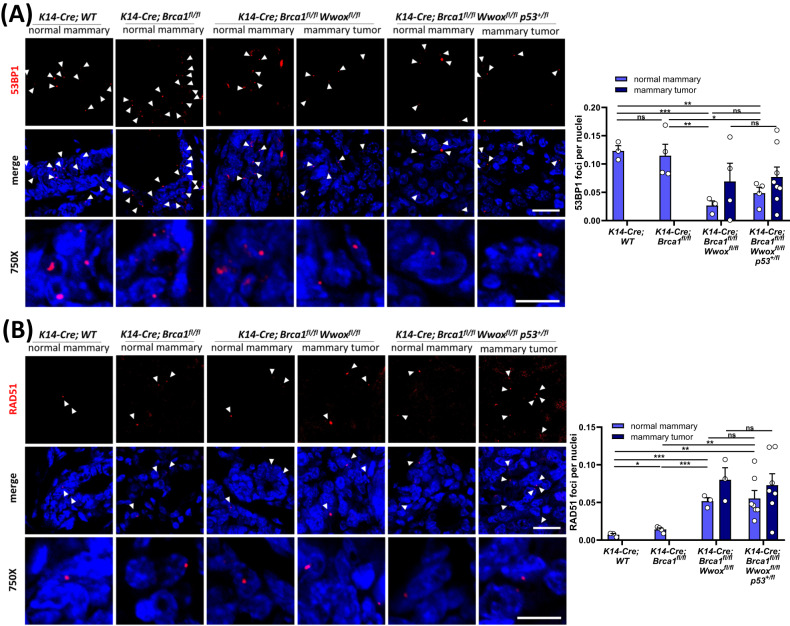
Combined loss of *Wwox* and *Brca1* redirects the DSB repair to NHEJ in vivo. **A** Left- Endogenous NHEJ DSB repair marked by 53BP1 foci in normal mammary or mammary tumors from: *K14-Cre;WT* (*n* = 3), *K14-Cre;Brca1*^*fl/fl*^ (*n* = 4), *K14-Cre;Brca1*^*fl/fl*^*Wwox*^*fl/fl*^ (*n* = 3) and *K14-Cre;Brca1*^*fl/fl*^*Wwox*^*fl/fl*^*Trp53*^+/fl^ (*n* = 8) mice. Arrow heads marking the foci. Merge- 160X scale- 10 µm, 750X scale- 20 µm. Right- quantification of 53BP1 foci per nuclei, three 40× fields were quantified from each mouse. Statistical analysis by *t*-test, *P* value < 0.05, error bars representing SEM. *P* value between normal and mammary tumor- not significant. **B** Left- Endogenous HDR DSB repair marked by RAD51 foci in normal mammary or mammary tumors from: *K14-Cre;WT* (*n* = 3), *K14-Cre;Brca1*^*fl/fl*^ (*n* = 4), *K14-Cre;Brca1*^*fl/fl*^*Wwox*^*fl/fl*^ (*n* = 3) and *K14-Cre;Brca1*^*fl/fl*^*Wwox*^*fl/fl*^*Trp53*^*+/fl*^ (*n* = 8). Arrow heads marking the foci. Merge- 160× scale- 10 µm, 750× scale- 20 µm. Right- quantification of RAD51 foci per nuclei, three 40X fields were quantified from each mouse. Statistical analysis by *t*-test, *P* value < 0.05, error bars representing SEM. *P* value between normal and mammary tumor- not significant.

Given the effect of combined deletion of *Wwox* and *Brca1* on mammary tumor formation and the known effect of WWOX/BRCA1 interaction on DNA repair pathway choice in vitro [17, 38, 39], we next set to examine which DSB repair pathway is favored in tumors of *K14-Cre;Brca1*^*fl/fl*^*;Wwox*^*fl/fl*^ mice. To test this in vivo, immunostaining of p53-binding protein 1 (53BP1), a NHEJ repair marker, RAD51, a surrogate marker for HDR, and γH2AX, marking DSBs, was performed and quantified as detailed in the Methods sections. Mammary tumors from *K14-Cre;Brca1*^*fl/fl*^*;Wwox*^*fl/fl*^ and *K14-Cre;Brca1*^*fl/fl*^*;Wwox*^*fl/fl*^*;Trp53*^*+/fl*^ mice exhibited elevated number of 53BP1, RAD51 and γH2AX foci per nuclei compared to their normal mammary (Fig. 2A, B and Supplementary Fig. 1). We did not observe a significant difference in these DNA damage marker’s foci number between the two mouse models; neither in the mammary tumors nor in the normal mammary epithelium. These results suggest an intact DDR signaling in these tumors and that the DSB repair pathway choice was not affected upon depleting one *Trp53* allele in *K14-Cre;Brca1*^*fl/fl*^*;Wwox*^*fl/fl*^ tumors.

We next compared the normal mammary tissue of *Brca1*^*fl/fl*^ mice to those of *K14-Cre;Brca1*^*fl/fl*^*;Wwox*^*fl/fl*^ and *K14-Cre;Brca1*^*fl/fl*^*;Wwox*^*fl/fl*^*;Trp53*^*+/fl*^ mice and found a substantial decrease in foci of the NHEJ marker 53BP1, in the normal mammary tissue lacking *Wwox* (Fig. 2A). On the other hand, when comparing RAD51 foci between the genotypes, we observed that loss of WWOX led to more abundant foci in the normal mammary cells (Fig. 2B). We also detected a slight increase in the RAD51 foci per nuclei in mammary epithelia of *K14-Cre;Brca1*^*fl/fl*^ mice compared to WT, likely suggesting impaired HDR repair (lacking BRCA1), or other RAD51-dependent repair mechanisms. These results correspond with previous in vitro findings [17, 38, 39], demonstrating that WWOX’s presence directs the DSB repair towards NHEJ, while its depletion results in compromised NHEJ repair and more HDR, even in the absence of BRCA1.

### WWOX overexpression in *BRCA1* wild-type TNBC cells is associated with elevated NHEJ

**Fig. 3:**
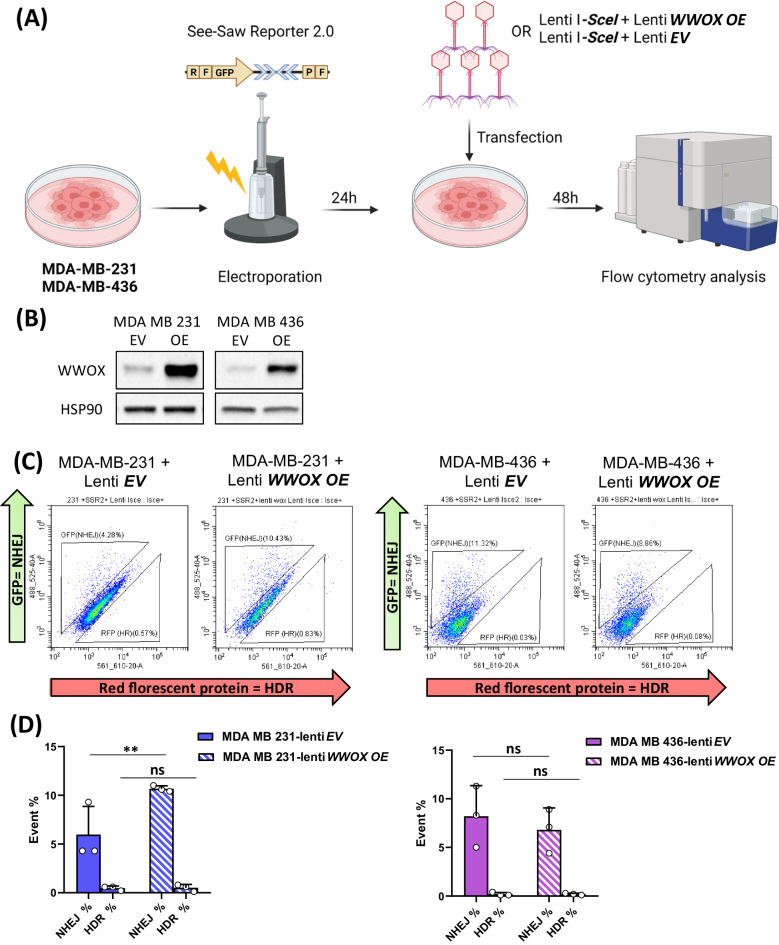
WWOX expression in *BRCA1 WT* TNBC cells elevates NHEJ as a response to direct DSB. **A** In vitro experimental design. MDA-MB-231 or MDA-MB-436 TNBC cell line were electroporated with the See-Saw Repoter 2.0 system plasmid, 24 h later, a lenti virus expressing the systems endonuclease, *IsceI*, was added in addition to lenti- *WWOX OE* or lenti- empty vector (*EV*). 48 h later the cells were analyzed via flow cytometry. **B** WWOX immunoblot of MDA-MB 231 and MDA-MB 436 with empty vector (EV) and *WWOX* overexpression (*OE*). HSP90 as a house keeping gene. **C** Flow-cytometry scatter plot of the MDA-MB-231 or MDA-MB-436 with lenti *EV* or lenti *WWOX OE* and the See-Saw system. Living cells, expressing *IsceI*, presenting HDR marked by RFP or NHEJ repair marked by GFP. **D** Quantification graph of the NHEJ and HDR cell populations percentages from the flow-cytometry results. three experimental repeats, statistical significance calculated by *t-*test, *P*-value < 0.01, error bars representing SEM.

The preceding results suggest that *Brca1* and *Wwox* cooperate in vivo to regulate DSB repair. To learn more about the role of WWOX and BRCA1 in immediate response to DSB repair of human TNBC, we used MDA-MB-231 and MDA-MB-436 cell lines. MDA-MB-231 harbors *BRCA1* WT with low protein expression levels of WWOX, while MDA-MB-436 carries a 5396 + 1 G > A mutation in the splice donor site of exon 20 of *BRCA1* [43] and express low protein levels of WWOX, as well. Using the recently developed SeeSaw Reporter 2.0 [44], we tested HDR and NHEJ repair outcome in these human TNBC cells in vitro. In this system, a double-strand break is generated by *ISceI* which can be alternatively repaired by homology-independent or -dependent mechanisms, leading to the accumulation of distinct fluorescent protein; RFP (red) for HDR and GFP (green) for NHEJ repair. Expression of the system’s endonuclease, *ISceI*, is tracked by BFP [42]. Twenty-four hours after electroporation of the SeeSaw reporter into the two human TNBC cell lines, *ISceI* together with *WWOX* or *EV* were introduced and 48 h later cells were analyzed via flow cytometry (Fig. 3A). The levels of WWOX were detected by western blot from lysates made of the same cells that were analyzed via flow cytometry (Fig. 3B). In MDA-MB-231 cells, *ISceI* + *WWOX* overexpressing (*OE*) cells exhibited a significant elevation in NHEJ repair (GFP+ cells) compared to *ISceI* + *EV* cells, as expected, due to WWOX’s role in NHEJ repair (Fig. 3C, D- left panels).

Surprisingly, MDA-MB-436 cells expressing WWOX didn’t display any significant change in the DSB repair pathways as compared to control (Fig. 3C, D- right panels). In both cell lines, no significant difference in the HDR levels was found between the *WWOX* overexpressing cells and their control. Altogether, these data imply that in human TNBC cells in vitro, WWOX shifted the DSB repair pathway toward NHEJ in the presence of BRCA1.

### *WWOX* overexpression elevates NHEJ and reduces HDR in TNBC cells upon exposure to DNA damaging agents

**Fig. 4:**
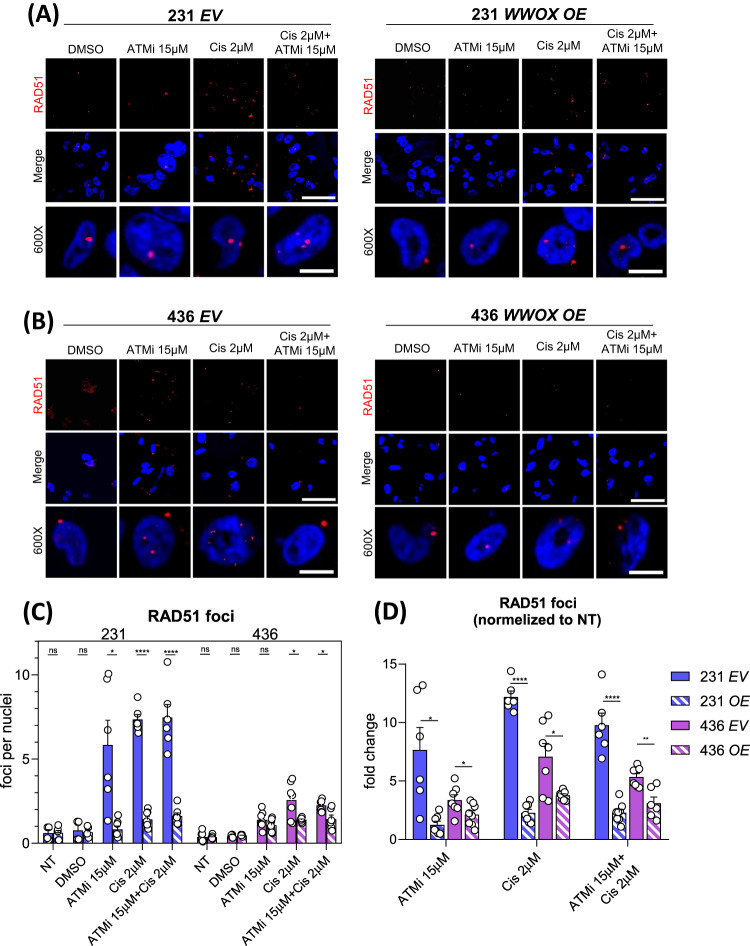
Loss of *WWOX* significantly reduces HDR repair, especially when BRCA1 is present. **A** HDR DSB repair marked by RAD51 foci in MDA-MB-231 TNBC cell line (sufficient BRCA1) with *EV* (left) or *WWOX OE* (right), with or without DNA damage agents (ATMi, cisplatin). **B** HDR DSB repair marked by RAD51 foci in MDA-MB-436 TNBC cell line (mutated *BRCA1*) with *EV* (left) or *WWOX OE* (right), with or without DNA damage agents (ATMi, Cisplatin). Scale: 120X-100uM, 600×-20uM. **C** Quantification of RAD51 foci per nuclei in MDA-MB-231 or 436 with *EV* or *WWOX OE*. At least 5 fields were quantified from each cell line and treatment. Statistical analysis by t-test, error bars representing SEM. **D** Fold change of RAD51 foci per nuclei treatment/ control of each of the cell lines. Statistical analysis by t-test, error bars representing SEM.

We next set to test how MDA-MB-231 and MDA-MB-436 cells expressing WWOX respond to DNA damage induction. Since platinum-based chemotherapies are often used in treatment of TNBC [45], we examined the effect of cisplatin treatment on empty vector (*EV*) or *WWOX* overexpressing (*OE*) TNBC cells. In addition, we examined sensitivity of these cells to ATM inhibitor (ATMi) as WWOX and ATM have been physically and functionally linked [34, 36]. To this end, MDA-MB-231 and MDA-MB-436 cells with *EV* and *WWOX OE* were treated with cisplatin (2 µM), ATMi (15 µM) or both for 24 hr, then immunostained for HDR and NHEJ DSB markers. We found that MDA-MB-436 cells treated with cisplatin and ATMi resulted in low levels of RAD51 foci, less than 5 foci per cell (Fig. 4B), likely due to their BRCA1 status [46, 47]. More interestingly, when comparing EV to *WWOX* OE cells, there was a significant reduction in the number of RAD51 foci per nuclei, especially in MDA-MB-231 cells (Fig. 4A–D). No synergistic effects were observed when using cisplatin and ATMi combined treatment. When normalizing the number of RAD51 foci per cell to their relative control without treatments, the same trends were preserved (Fig. 4D).

**Fig. 5:**
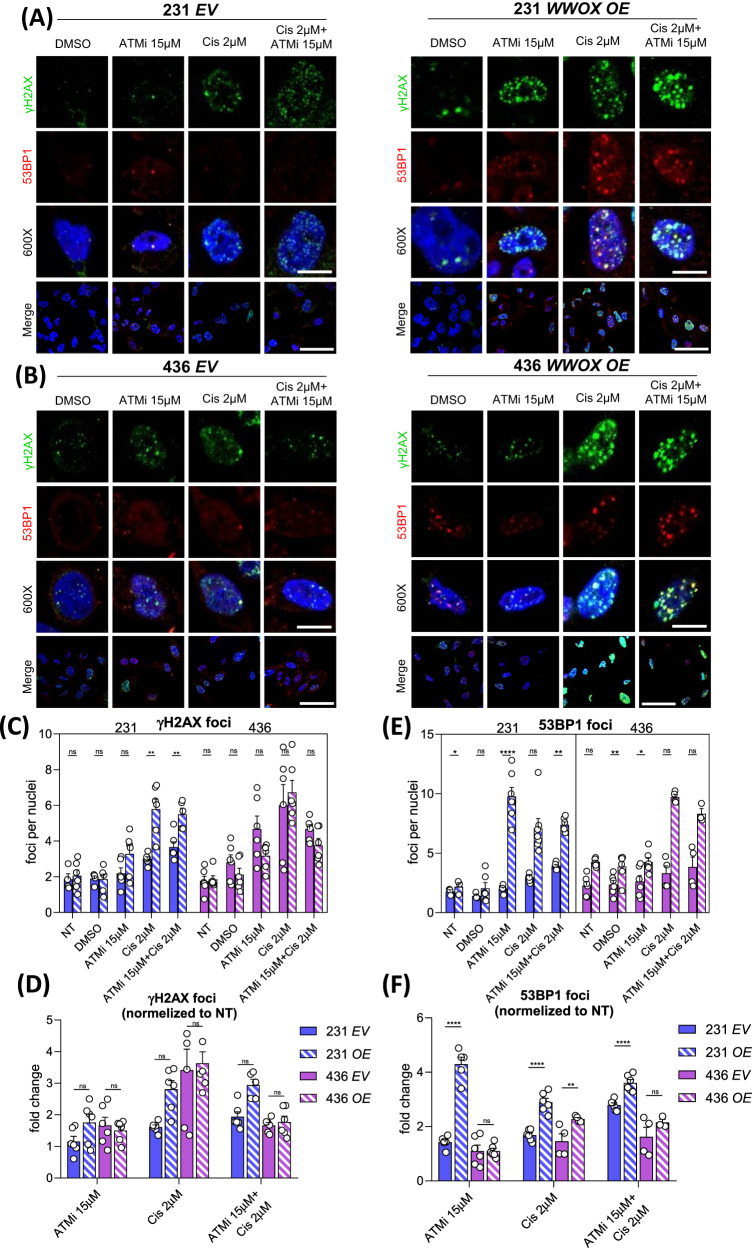
Loss of *WWOX* doesn’t lead to more DNA damage but does increase NHEJ. **A** DNA DSB (γH2AX) and NHEJ repair (53BP1) foci in MDA-MB-231 TNBC cell line with *EV* (left) or *WWOX OE* (right), with or without DNA damage agents (ATMi, cisplatin). **B** DNA DSB (γH2AX) and NHEJ repair (53BP1) foci in MDA-MB-436 TNBC cell line (mutated *BRCA1*) with *EV* (left) or *WWOX OE* (right), with or without DNA damage agents (ATMi, cisplatin). Scale: 120×-100μM, 600×-20μM. Quantification of γH2AX (**C**) and 53BP1 (**E**) foci per nuclei in MDA-MB-231 or 436 with *EV* or *WWOX OE*. At least 5 fields were quantified from each cell line and treatment, error bars representing SEM. Fold change of γH2AX (**D**) and 53BP1 (**F**) foci per nuclei treatment/ control of each of the cell lines. Statistical analysis by *t*-test, error bars representing SEM.

We next stained and quantified 53BP1 in MDA-MB-231 and MDA-MB-436 cells. In contrast to the trend observed in RAD51 foci, 53BP1 foci per cell were more abundant in the cells overexpressing *WWOX* compared to *EV* (Fig. 5A–F). The difference again was more prevalent in MDA-MB-231 cells, raising the possibility that the DSB repair pathway choice under different *WWOX* levels is partially BRCA1-dependent. Based on the observed difference between *EV* and *OE* in *BRCA1*-mutated MDA-MB-436 cells, it is plausible that BRCA1 alone may not be the only player responsible for WWOX-mediated NHEJ, indicating the involvement of other factors or pathways in this repair mechanism. On another note, when comparing the number of 53BP1 foci in MDA-MB-231 cells treated with cisplatin or combined treatment with ATMi, we could see an increase in the latter treatment both in *EV* and *WWOX OE*. On the other hand, in MDA-MB-436 there was no significant difference between the two treatments. This implies that inhibition of ATM causes NHEJ stimulation in a BRCA1-related manner.

Additionally, we examined γH2AX foci and observed an increase in DSBs, as anticipated. Nevertheless, we did not detect any notable variations in the levels of damage among the distinct cell lines or genotypes (Fig. 5A–D), suggesting that the differences in the number of RAD51 and 53BP1 foci are not due to the number of DSBs. Altogether, our findings suggest that WWOX expression promotes DSB repair via the NHEJ pathway rather than HDR.

## Discussion

**Fig. 6:**
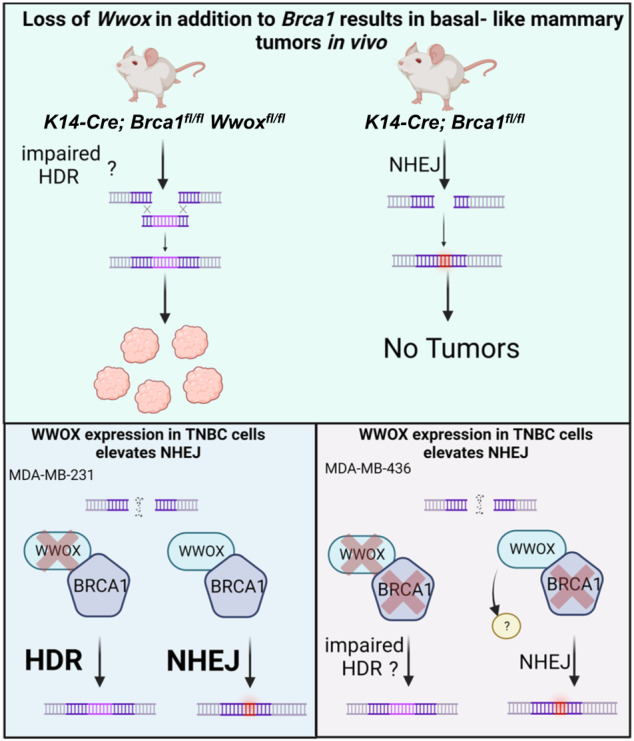
Graphical abstract summarizing the role of *WWOX* in vivo and in vitro. *K14-Cre;Brca1*^*fl/fl*^*Wwox*^*fl/fl*^ mice harboring impaired HDR and insufficient NHEJ result in mammary tumor development, as compared to tumor-free *K14-Cre; Brca1*^*fl/fl*^ mice relying on NHEJ. MDA-MBA-231 cells with *WT BRCA1*, exhibit high levels of HDR, while 231-overexpressing *WWOX* shifts DSBs repair to NHEJ. MDA-MBA-436 with mutant *BRCA1*, exhibit low levels of HDR, while 436-overexpressing *WWOX* shifts DSBs repair to NHEJ mediated by unknown DDR player.

Loss of BRCA1 and WWOX has been previously linked with TNBC development. Given that both tumor suppressors play a role in repairing DSBs, we aimed to test their functional interplay both in vitro and in vivo. We demonstrated that combined deletion of murine *Wwox* and *Brca1* in mammary gland epithelium resulted in accelerated BLBC-like tumors. Characterization of DSB repair in these tumors lacking WWOX and BRCA1 expression revealed increased RAD51 and decreased 53BP1 foci relative to normal adjacent tissues suggesting that absence of WWOX redirects DSBs repair pathway away from NHEJ. These results were further confirmed in vitro using human TNBC cells and HDR/NHEJ reporter system (Fig. 6-graphical abstract).

WWOX has been primarily mapped to fragile site FRA16D in breast cancer due to its common deletion and alteration [48]. The FRA16D locus is a chromosomal region on chromosome 16q23.2 that is prone to DNA breakage and rearrangements, particularly in cancer cells undergoing replication stress [34]. Several studies have shown that WWOX is frequently subjected to genetic alterations, such as deletions, mutations, and loss of expression, in various cancers, including breast cancer [27, 28, 49]. We and others have recently shown that gene products of common fragile sites can have direct roles in DDR [34, 36, 37, 50, 51]. In fact, WWOX expression is induced early upon DNA damage contributing to proper DDR signaling [36, 37]. Therefore, loss of WWOX makes cells more susceptible to genomic instability.

Loss or reduced expression of WWOX has been associated with breast cancer development and progression [23, 25, 52–58]. As a tumor suppressor gene and a scaffold protein, WWOX plays a crucial role in regulating multiple cellular processes, including cell growth, apoptosis, DNA repair, and maintenance of genomic stability [34]. WWOX inactivation can hence contribute to the disruption of these processes, promoting tumorigenesis in breast cancer. Recent genomic studies have identified germline biallelic aberrations of *WWOX* in patients with multiple primary cancers including breast cancer. Interestingly, co-occurrence of *WWOX* mutations has been observed with genetic variants in genes involved in DNA repair, such as *BRCA2, TP53, CHK2* and *RAD51D* [59, 60].

We have shown previously that WWOX cooperates with p53 to antagonize mammary tumor formation [27, 59]. In human breast cancer, there is evidence of a correlation between nuclear BRCA1 and WWOX expression [52]. However, a detailed study investigating the expression of WWOX in various BRCA1 variants and mutations doesn’t yet exist. Our findings here show for the first time that in *K14-Cre* conditional murine model, combined deletion of *Wwox* and *Brca1* synergizes to accelerate mammary tumor formation in vivo. Individually, the loss of either *Wwox* or *Brca1* in the *K14-Cre* model may not be sufficient to initiate tumor formation. This implies that there is cooperation between WWOX and BRCA1 in suppressing tumorigenesis. This also suggests that both WWOX and BRCA1 play crucial roles in maintaining the normal function of cellular processes, such as DNA integrity, that prevent tumor development. When both genes are simultaneously deleted, there is a synergistic effect that impairs this integrity, leading to accelerated tumor development. The interaction between WWOX and BRCA1 may involve shared or complementary functions in regulating key pathways involved in cell growth control, DNA repair, and maintenance of genomic stability. Loss of either gene alone may be compensated for by the remaining intact gene or by alternative pathways. However, the simultaneous loss of both genes likely disrupts these compensatory mechanisms, leading to an increased susceptibility to tumor formation. Deletion of one p53 allele further accelerates tumor formation mediated by loss of *Wwox* and *Brca1*, further demonstrating the significance of this triad in breast cancer development.

How WWOX expression inhibits HDR is not well understood. Clear evidence proposed by our data suggests that WWOX expression favors NHEJ rather than HDR. A recent study has revealed that WWOX negatively regulates HDR activity in Hela cells [18]. Taouis et al. have identified MERIT40 as a novel molecular partner of WWOX and demonstrated that the interaction between WWOX and MERIT40 hinders the ability of MERIT40 to bind to Tankyrase. Tankyrase plays a positive role in NHEJ by stabilizing the kinase DNA-PK. The inhibition of Tankyrase-MERIT40 interaction by WWOX suppresses the enhancing function of this complex in HDR, while facilitating the role of Tankyrase in NHEJ. In contrast, a previous paper by Abu-Odeh and colleagues, has shown that WWOX enhances HDR in U2OS cells [36]. We assume that the different cell lines and reporter systems as well as the cell-specific behaviors could contribute to the varying outcomes between this article and the current one. Our findings, both in vivo and in vitro are in alignment with a prior study conducted by Park et al. [17]. Their research revealed heightened RAD51 levels in WWOX-negative cells and established a link between the absence of WWOX and enhanced DNA end resection. This premature resection can lead to increased HDR before the S phase, potentially undermining the precision of HDR and contributing to mutagenic outcomes, genome instability and risk of tumorigenesis. Our mice with dual deficiencies in WWOX and BRCA1 exhibit increased levels of RAD51, indicative of heightened HDR. However, unlike mice with BRCA1 deficiency alone, the double knockout mice developed tumors. This observation implies that the double KO mice may indeed have elevated RAD51 levels and increased homology-directed repair, but its effectiveness appears compromised. Whether this previously discovered mechanism in vitro is also true for our in vivo system will still need to be answered. Our data reveal that in the absence of BRCA1 and WWOX, RAD51 and 53BP1 foci are still detected. It is plausible that combined ablation of *Wwox* and *Brca1*, in our genetically engineered mice, results in impaired HDR and NHEJ. The presence of RAD51 in mammary cells with deletion of *Brca1*, or in human MDA-MB-436 cells with a loss of function mutation in *BRCA1*, can prove that RAD51 might participate in different pathways other than HDR. Indeed, RAD51 has been found to play a role in less efficient repair mechanisms of DSBs such as interstrand cross-link repair or break-induced replication [60–63] that can result in serious consequences for DNA integrity. Furthermore, RAD51 is found to be overexpressed in several types of cancer, and implicated in resistance to chemotherapy [64–66], in contrast to the other components of the HDR pathway. It is possible that the non-canonical properties of RAD51 are manifested when BRCA1 is absent, together with the genomic instability that is enhanced upon absence of WWOX, causes extensive DNA damage, supporting cancer development, and resistance to treatments. Our data also revealed treatment resistance in human MDA-MB-231 and MDA-MB-436 cells lacking WWOX (not shown). Following ATMi treatment, TNBC cells lacking WWOX express less sub G1 population in cell- cycle flow-cytometry compared to *WWOX* overexpressing counterparts (not shown).

Similarly, the persistence of 53BP1 foci in BRCA1 mutant cells indicates that the NHEJ pathway is still functional to some extent. While BRCA1 is not directly involved in NHEJ, it is possible that other proteins and factors can contribute to the recruitment and activation of 53BP1 in these cells. We acknowledge that while 53BP1 is commonly used as an indicator of NHEJ, it might not provide a direct measure of NHEJ activity. Nevertheless, there are several lines of evidence supporting the use of 53BP1 as a NHEJ marker [17, 67, 68]. For precise assessment of NHEJ, additional investigation will be required. Overall, the presence of RAD51 and 53BP1 foci in WWOX and BRCA1 mutant cells suggests a dynamic interplay between different DNA repair pathways and compensatory mechanisms. This highlights the complex nature of DNA repair and the ability of cells to utilize alternative pathways when the primary repair mechanisms are compromised.

In summary, our study revealed that WWOX and BRCA1 loss led to the development of basal-like mammary tumors and impaired DSB repair. WWOX promoted NHEJ repair in cells with wild-type BRCA1. Additionally, TNBC cells dependent on WWOX-mediated NHEJ repair showed increased sensitivity to DNA damaging agents. These findings suggest that relying solely on HDR for DSB repair may be insufficient in mammary cells, leading to tumorigenesis and DNA damage resistance. WWOX plays a crucial role in the choice of DSB repair pathway in mammary cells, highlighting its significance as a tumor suppressor in breast carcinogenesis.

## Methods

### Mice

*Brca1*^*tm2Cxd*^/Nci [*Brca1*^*Δ11*^*, Strain Number: 01XC8*] mice were ordered from NCI Mouse Repository. These mice carry *Loxp* sites around exon 11 of *Brca1* (*Brca1*^*Δ11*^ *or Brca1*^*fl/fl*^), which encodes 60% of the protein [69]. *Brca1*^*fl/fl*^ mice were bred with *K14-Cre;* For the sake of simplicity in terminology, we referred to progeny of *K14-Cre*;*Brca1*^*fl/fl*^ mice as Brca1 KO. *Wwox*^*fl/fl*^ mice to generate double conditional knockout (DKO) mice: *K14-Cre*;*Brca1*^*fl/fl*^*;Wwox*^*fl/fl*^. DKO mice were also bred with *Trp53*^*+/fl*^ to generate *K14-Cre*;*Brca1*^*fl/fl*^*;Wwox*^*fl/fl*^;*Trp53*^*+/fl*^. Mammary tissues or tumors were fixed in 4% formaldehyde and processed for H&E staining and immunohistochemistry (IHC). Our mice were handled in the specific pathogen free (SPF) animal facilities of the Hebrew university according to the ethical standards approved by the Institutional Animal Care and Use Committee.

### Cell culture

MDA-MB-231 (CVCL_0062) and MDA-MB-436 (CVCL_0623) cell lines were grown in DMEM (Gibco) supplemented with 10% FBS (Gibco), glutamine, and penicillin/streptomycin (Biological Industries). All cells were grown in 37 °C with 5% CO_2_. Cells were routinely authenticated and confirmed as Mycoplasma-free, and cell aliquots from early passages were used. Stable clones for *Wwox* overexpression were produced using a lentiviral vector containing either empty vector (*EV*) or *WWOX* overexpression (*WWOX OE*). Clones were selected using 2 mg/mL G418 (Gibco 11811031).

### Histology and Immunohistochemistry

Tissues were fixed with 4% formalin, then 70% ethanol and processed. Paraffin-embedded tissue sections were deparaffinized and rehydrated, then stained with H&E for histological observation and diagnosis. For IHC, paraffin-embedded tissues were deparaffinized, followed by antigen retrieval using 25 mM citrate buffer (pH 6) in a pressure cooker. Then The sections were left to cool for 25 min, followed by blocking of the endogenous peroxidase with 3% H2O2 for 15 min. To reduce nonspecific binding of the primary antibody, tissues were blocked with blocking solution (CAS Block, Invitrogen, San Diego, CA), followed by incubation with the primary antibody overnight at 4 °C: Rb polyclonal anti WWOX [70] (1:8,000); Rb anti CK14 (ab181595, 1:2000); Rb anti ER (1:350, Sc-543); Rb anti p53 (1:400, NCLP53CM5p). Sections were washed 3 times with TBST and incubated with secondary HRP anti-rabbit IgG antibody for 30 min. After additional washes using TBST, the reaction was then performed using a DAB peroxidase kit (Vector Laboratories, SK-4100, Mowry Ave Newark, United State), followed by hematoxylin stain for 45 s as a counterstain.

### Immunofluorescence

Tissues were fixed in 4% formalin, then 70% ethanol and processed. Paraffin-embedded tissue sections were deparaffinized and rehydrated. Antigen retrieval was performed in 25 mM sodium citrate buffer PH 6.0 using pressurized chamber for 2.5 min. The sections were then incubated with blocking buffer (5% goat serum+0.5% BSA in PBT) for 1 h to reduce nonspecific binding followed by incubation with the antibodies overnight at 4 °C- Rb anti γH2AX (1:200, #9718), Rb anti-RAD51 (1:200, GTX100469), Rb anti-53BP1 (1:200, ab36823), Rb anti BRCA1 (1:100, sc-646). Slides were subsequently washed with PBS, incubated with secondary anti-Rabbit Alexa fluor 647 (1:300, ab150079) for one hour, in addition to Hoechst nuclear counter stain. Finally, slides were mounted by Dako’s Fluorescence Mounting Medium.

Cells were seeded on round slide coverslips in 12-well plates and treated with DMSO 1:1000, ATMi 15 µM (KU55933, sigma), 2 µM cisplatin (Courtesy of Hadassah Hospital) or combined ATMi 15 µM and 2 µM cisplatin; doses were based on previous literature [71, 72] and our own optimization. 24 hours later, cells were fixed in 4% PBS buffered formaldehyde and permeabilized with 0.05% Triton X-100 at room temperature. Cells were then incubated in blocking buffer (5% goat serum+0.5% BSA in PBT) for 1 hour, followed by primary antibodies overnight at 4 °C: mouse anti-γH2AX (1:1000, ab26350), Rb anti-RAD51 (1:200, GTX100469), Rb anti-53BP1 (1:200, ab36823). After PBS washes, slides were incubated with secondary antibodies- anti-Rabbit Alexa fluor 647 (1:300, ab150079) anti-Mouse Alexa fluor 488 (1:1000, A11029) for 1 hour in addition to Hoechst nuclear counter stain. Slides were mounted by Dako’s Fluorescence Mounting Medium. For the DNA damage markers staining of the mice’s normal mammary and tumors, at least four 40X fields were imaged, then quantified manually by counting the foci, and the nuclei. For the MDA-MB-231 and MDA-MB-436 cells immunofluorescence, the slides have been scanned by a digital slide scanner (3Dhistech); at least four 40X fields were selected and quantified by Qupath software [73] for the number of foci and nuclei.

### Electroporation and Lenti-Transduction

For electroporation, NEON transfection system was used. Cells (5*10^7^ cells/ml) were washed with PBS then resuspended with resuspension buffer R together with 6 μg DNA from each plasmid. Cells were chocked using the electroporation device, then were seeded for 24 h in medium containing serum and supplements without antibiotics. For Lentiviral transduction, 1 ml of medium with suspended cells was infected with 1 ml of medium containing the relevant Lenti-Virus + 1:1000 polybrene. To create stable clones the cells were later selected using 2 mg/mL G418 (Gibco 11811031).

### SeeSaw reporter (SSR 2.0)

Cells were electroporated with the See-Saw Repoter 2.0 system plasmid, 24 h later, a lenti virus expressing the systems endonuclease, *IsceI*, was added in addition to lenti- *Wwox* overexpression (*Wwox OE*) or lenti- empty vector (*EV*). A double-strand break is created *IsceI* endonuclease and can be alternatively repaired by homology-independent or -dependent mechanisms, leading to the accumulation of distinct fluorescent proteins; RFP (red) for HDR and GFP (green) for NHEJ repair. Expression of the system’s endonuclease, *ISceI*, is reported by BFP [45]. 48 h after addition of the lenti- viruses the cells were analyzed via flow cytometry.

### Flow cytometry

Cells were tripsinized and centrifuged, then their pellets were fixed using 1 ml of methanol-PBS (9:1) overnight in −20 °C. Cells were then centrifuged and resuspended with FACS buffer (PBS, 2% fetal bovine serum, 1 mM EDTA, 0.1% sodium azide) and filtered by mesh. Flow cytometry readings were done by Beckman Coulter CytoFlex. GFP was excited using a 488 nm laser and acquired with a 525-40 filter, RFP was excited using a 561 nm laser and acquired with a 610-20 filter, BFP was excited on a 405 nm laser and acquired with a 450–50 filter.

### Supplementary information

Supplemental Figure 1

Supplemental Figure 2

Supplementary Figure Legends

Raw data Western blot

## Data Availability

Raw data are available upon reasonable request.
