# Supplementary figures and images for: Unveiling the relationship between WWOX and BRCA1 in mammary tumorigenicity and in DNA repair pathway selection
Source: Cell Death Discov. 2024 Mar 18;10:145. doi: 10.1038/s41420-024-01878-8 (PMC10948869; doi:10.1038/s41420-024-01878-8)

## Slide 1
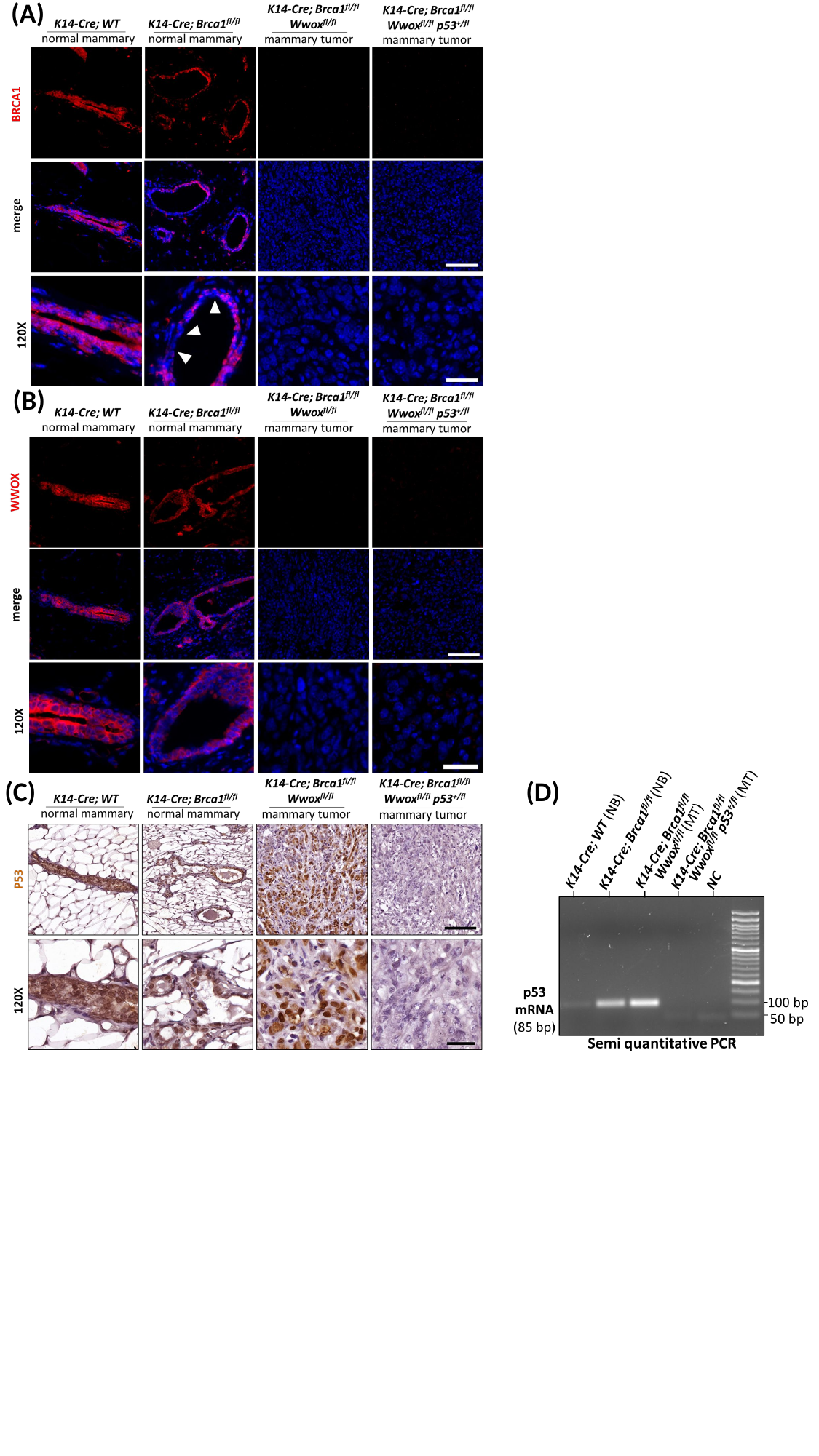

(A)
(B)
(C)
(D)

Supplement: Supplementary file 1 — Supplemental Figure 1 [file 41420_2024_1878_MOESM1_ESM.pptx]

## Slide 1
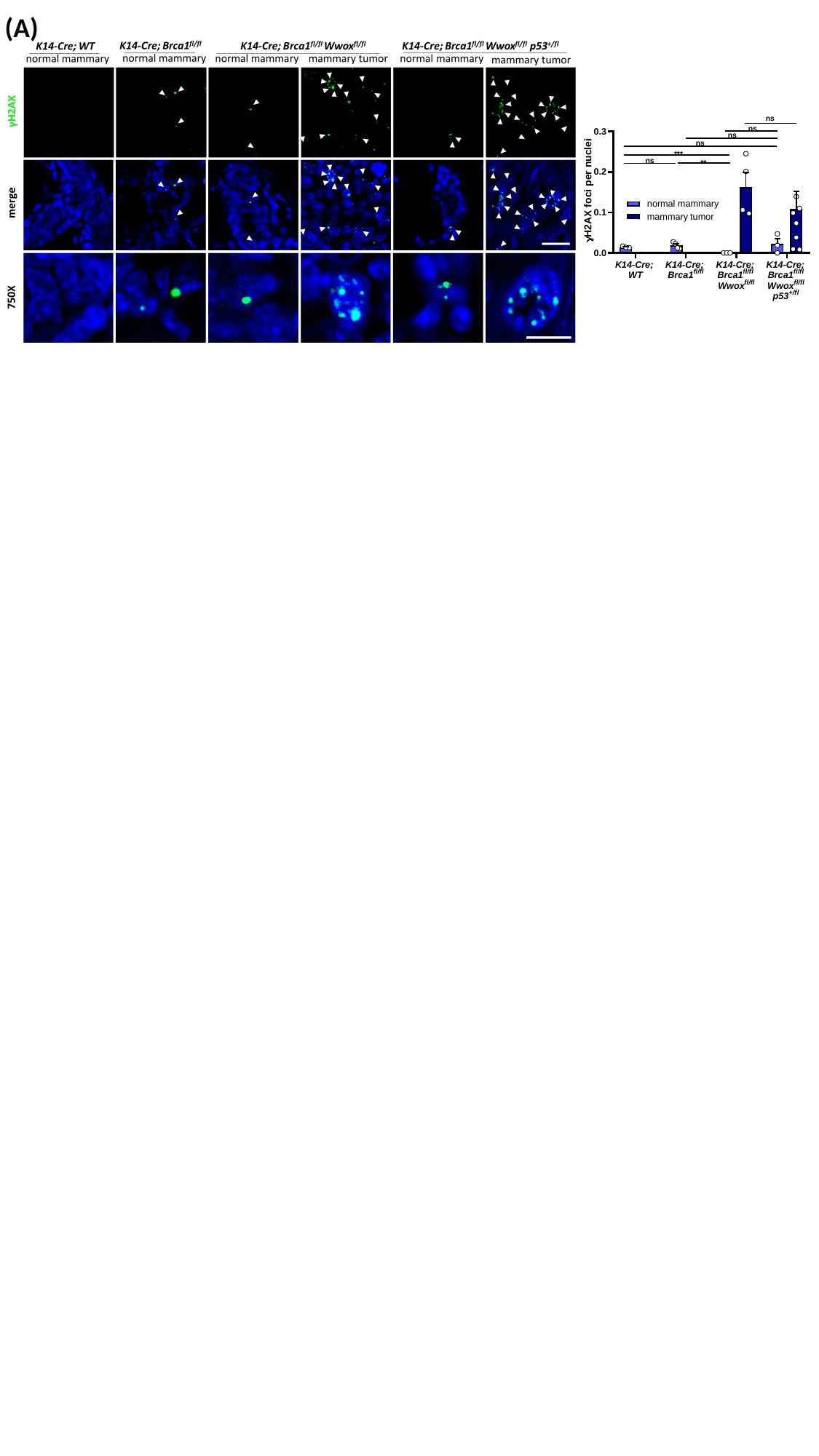

(A)

Supplement: Supplementary file 2 — Supplemental Figure 2 [file 41420_2024_1878_MOESM2_ESM.pptx]
