# Supplementary material for: Unveiling the relationship between WWOX and BRCA1 in mammary tumorigenicity and in DNA repair pathway selection
Source: Cell Death Discov. 2024 Mar 18;10:145. doi: 10.1038/s41420-024-01878-8 (PMC10948869; doi:10.1038/s41420-024-01878-8)
Supplement: Supplementary file 3 — Supplementary Figure Legends [file 41420_2024_1878_MOESM3_ESM.docx]

**Supplementary Material Legends:
Sup. Fig. 1**BRCA1, WWOX and P53 levels in mice mammary tumors and normal epithelium.
(A) BRCA1 staining in normal mammary (*K14-Cre;WT* and *K14-Cre;Brca1^fl/fl^* mice), or mammary tumors (*K14-Cre;Brca1^fl/fl^Wwox^fl/fl^* and *K14-Cre;Brca1^fl/fl^Wwox^fl/fl^Trp53^+/fl^* mice). Arrow heads marking negative cells. 40X scale- 100µm ,120X scale- 30µm.
(B) WWOX staining in normal mammary (*WT* and *Brca1^fl/fl^* mice), or mammary tumors (*K14-Cre;Brca1^fl/fl^Wwox^fl/fl^* and *K14-Cre;Brca1^fl/fl^Wwox^fl/fl^Trp53^+/fl^* mice). 40X scale- 100µm ,120X scale- 30µm.
(C) P53 staining in normal mammary (*K14-Cre;WT* and *K14-Cre;Brca1^fl/fl^* mice), or mammary tumors (*K14-Cre;Brca1^fl/fl^Wwox^fl/fl^* and *K14-Cre;Brca1^fl/fl^Wwox^fl/fl^Trp53^+/fl^* mice). 40X scale- 100µm, 120X scale- 30µm.
(D) Semi quantitative PCR results for amplification of p53 mRNA (cDNA). NB- normal mammary, MT- mammary tumor.

**Sup. Fig. 2**Endogenous γH2AX stainings in mammary tumors and normal epithelium.
(A) Left- DSB marked by γH2AX foci in normal mammary or mammary tumors from: *K14-Cre;WT* (n=3), *K14-Cre;Brca1^fl/fl^* (n=4), *K14-Cre;Brca1^fl/fl^Wwox^fl/fl^* (n=3) and *K14-Cre;Brca1^fl/fl^Wwox^fl/fl^Trp53^+/fl^* (n=8). Arrow heads marking the foci. Merge- 160X scale- 10µm, 750X scale- 20µm. Right- quantification of γH2AX foci per nuclei, three 40X fields were quantified from each mouse. Statistical analysis by t-test, Pvalue>0.05, error bars representing SEM. Pvalue between normal and mammary tumor- not significant.
