# Supplementary material for: Unveiling the relationship between WWOX and BRCA1 in mammary tumorigenicity and in DNA repair pathway selection
Source: Cell Death Discov. 2024 Mar 18;10:145. doi: 10.1038/s41420-024-01878-8 (PMC10948869; doi:10.1038/s41420-024-01878-8)
Supplement: Supplementary file 4 — Raw data Western blot [file 41420_2024_1878_MOESM4_ESM.pptx]

## Slide 1
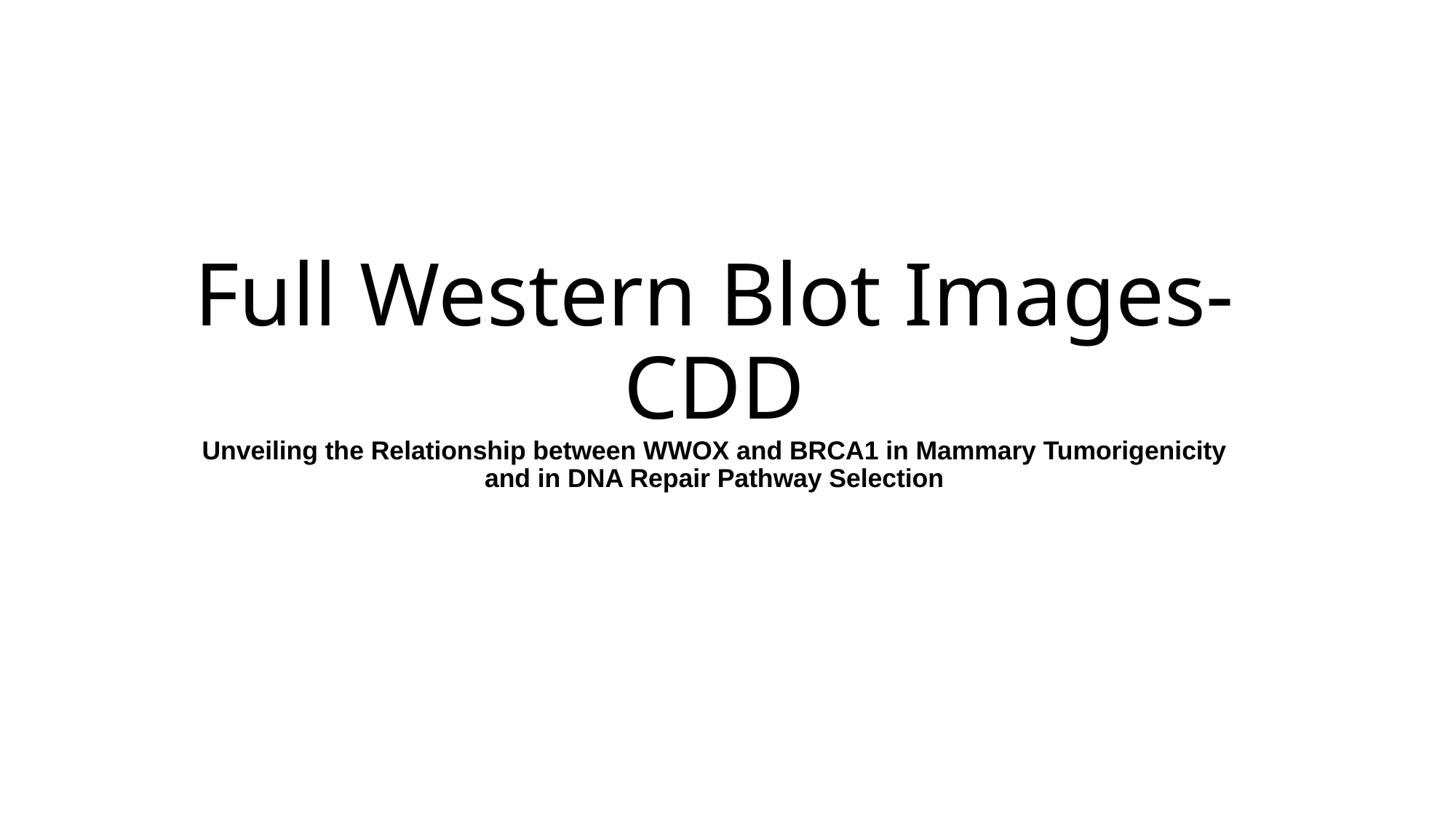

# Full Western Blot Images- CDD Unveiling the Relationship between WWOX and BRCA1 in Mammary Tumorigenicity and in DNA Repair Pathway Selection

## Slide 2
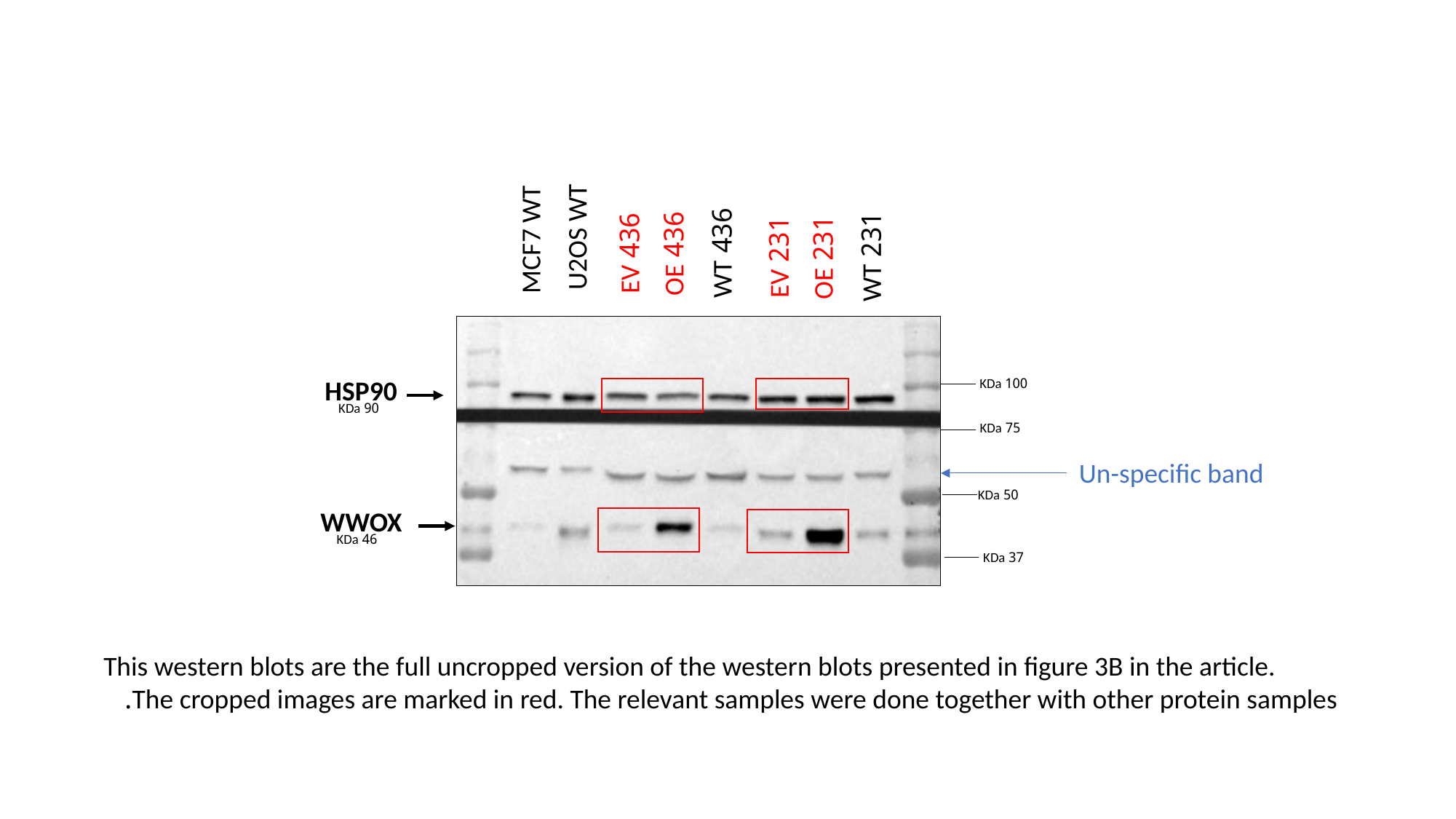

U2OS WT
MCF7 WT
436 WT
436 EV
436 OE
231 WT
231 EV
231 OE
HSP90
100 KDa
90 KDa
75 KDa
Un-specific band
50 KDa
WWOX
46 KDa
37 KDa
This western blots are the full uncropped version of the western blots presented in figure 3B in the article.
The cropped images are marked in red. The relevant samples were done together with other protein samples.
